# Early Combined SHP2 Targeting Reverses the Therapeutic Resistance of Vemurafenib in Thyroid Cancer

**DOI:** 10.7150/jca.83853

**Published:** 2023-05-29

**Authors:** Weike Ma, Mengran Tian, Linfei Hu, Xianhui Ruan, Wei Zhang, Xiangqian Zheng, Ming Gao

**Affiliations:** 1 Department of Thyroid and Neck Tumor, Tianjin Medical University Cancer Institute and Hospital, National Clinical Research Center for Cancer, Key Laboratory of Cancer Prevention and Therapy, Tianjin's Clinical Research Center for Cancer, Tianjin 300060, China.; 2 Tianjin Union Medical Center, No.190 Jieyuan Road, Hongqiao District, Tianjin 300121, China.; 3 School of Medicine, Nankai University, Tianjin 300071, China.

**Keywords:** Thyroid cancer, vemurafenib, BRAF inhibitor resistance, SHP2, combination strategy

## Abstract

The BRAFV600E mutation is the most common oncogenic mutation in thyroid cancer, suggesting an aggressive subtype of thyroid cancer and poor prognosis. Vemurafenib, a selective inhibitor of BRAFV600E, may provide therapeutic benefit in various cancers including thyroid cancer. However, the prevalence of drug resistance remains a challenge because of the feedback activation of the MAPK/ERK and PI3K/AKT pathways. In treating thyroid cancer cells with vemurafenib, we have detected reactivation of the MAPK/ERK signaling pathway as a result of the release of multiple receptor tyrosine kinases (RTKs) from the negative feedback of ERK phosphorylation. SHP2 is an important target protein downstream of the RTK signaling pathway. Decreasing it through SHP2 knockdown or the use of an inhibitor of SHP2 (SHP099) was found to significantly increase the early sensitivity and reverse the late resistance to vemurafenib in BRAFV600E mutant thyroid cancer cells. Overall, our findings suggest that blocking SHP2 reverses the reactivation of the MAPK/ERK signaling pathway caused by the activation of RTKs and improves the sensitivity of thyroid cancer to vemurafenib, which has potential implications for mechanism-based early combination strategies to treat thyroid cancer.

## Introduction

In previous studies on the molecular genetic mechanisms of thyroid cancer, BRAF mutations were found to be the most frequently occurring mutations detected in 60-70% of papillary thyroid cancer (PTC) and 41-45% of anaplastic thyroid cancer (ATC) 1-3. The results of a multicenter study showed that BRAF mutations were strongly associated with lymph node metastasis, extrathyroidal invasion, advanced stage III and IV disease, and disease recurrence 4. This makes BRAFV600E a prognostic molecular marker and a promising therapeutic target for thyroid cancer. Targeted therapies to inhibit BRAFV600E mutations have had initial success, but side effects and resistance to single-agent BRAFV600E inhibitors have often led to the termination of targeted therapy 5-7. However, the mechanisms causing adaptive resistance to BRAF inhibitors in thyroid cancer remain to be elucidated, and strategies to overcome drug resistance are urgently needed in clinical practice.

The mechanisms of resistance to BRAF inhibitors have been extensively studied with reported mechanisms including changes in key signaling pathways, changes in the tumor microenvironment, elevated ABC transporter proteins, downregulation of apoptotic signaling, and elevated levels of autophagy 8-13. Among reported mechanisms, the paradoxical activation of the mitogen-activated protein kinase (MAPK) pathway by RAF inhibitors has been cited several times in the article, so a more thorough description of this pathway is warranted. Generally, the MAPK signaling pathway is activated when extracellular mitogens bind to homologous receptors on the cell membrane, typically RTKs 14. Naturally, even though various ligands are able to bind homologous receptors in BRAFV600E cells, signaling downstream of the activated receptors is blocked due to strong ERK-dependent negative feedback regulation 15, 16. In this condition, RAS-GTP activity is low and BRAFV600E exists mainly as a monomer that is sensitive to BRAF inhibitor treatment. However, prolonged exposure of BRAFV600E cells to BRAF inhibitors is often accompanied by adaptive changes in signaling networks. Cancer cells can reactivate these signaling pathways through a number of molecular mechanisms, resulting in drug resistance 8, 17. Therefore, the use of BRAF inhibitors in combination with other drugs to prevent reactivation of the MAPK pathway may be an important means to address BRAF inhibitor resistance.

Given that RTKs are typical upstream receptors for MAPK signaling pathway activation, combining BRAF inhibitors with RTK inhibitors may be an important strategy to inhibit vemurafenib resistance. Previous studies found a central role for epidermal growth factor receptor (EGFR), mesenchymal-epithelial transition factor (MET) and insulin-like growth factor 1 receptors (IGFR1) in abolishing RAF inhibitor sensitivity in colorectal cancer and melanoma with BRAFV600E 18-22. However, the strategy of combining BRAF inhibitors with RTK inhibitors has some limitations, as different BRAFV600E cell lines can be rescued by different RTK ligands and multiple ligands may be responsible for the attenuation of RAF inhibitor sensitivity in individual BRAF mutant patients, so a single combination regimen is unlikely to be optimal for all patients 23. Protein tyrosine phosphatase 2 (SHP2), encoded by the PTPN11 gene containing Src homologue-2, is a 593 amino acid classical nonreceptor protein tyrosine phosphatase (PTP) 24. In cells, SHP2 acts downstream of various RTKs in the cytoplasm and is involved in many oncogenic cells signaling cascades (e.g., RAS-ERK, PI3K-AKT, and JAK-STAT) 25. SHP2 has emerged as a key regulator of RTKs and cytokine receptor signaling 26, 27. Furthermore, because of the overlap of SHP2 and RTKs signaling pathways, SHP2 inhibitors can be used in combination with kinase inhibitors thereby providing dual inhibition of the interconnected signaling pathways. This combination therapy may be more effective than monotherapy, both in terms of being less prone to resistance and in reversing acquired resistance to kinase inhibitors. SHP099 is a potent small molecule inhibitor of SHP2 that inhibits MAPK signaling and proliferation in RTK-dependent cells by directly targeting and inhibiting SHP2 28. In RTK-driven cancer cell lines, SHP099 inhibits MAPK pathway activity and suppresses malignant growth in vitro and in vivo tumor models 25, 28. However, its effect on thyroid cancer is unknown.

In the present study, we found that most of the RTK receptors and ligands were activated during the treatment of thyroid cancer cells with the BRAF inhibitor vemurafenib, while SHP2, a key protein downstream of the RTK signaling pathway, was also activated. As a result, the MAPK/RAS and PI3K/AKT signaling pathways were reactivated. In addition, we found that blocking the expression of SHP2 significantly improved the sensitivity of vemurafenib. And SHP099 in combination with vemurafenib was significantly superior to vemurafenib alone in terms of antitumor effects. Moreover, SHP099 was able to reverse the adaptive resistance of BRAFV600E mutant thyroid cancer cells to vemurafenib. These preliminary results suggest that the vemurafenib and SHP099 combination strategy may be a potential treatment for BRAFV600E mutant thyroid cancer.

## Materials and methods

### Cell culture and reagents

K-1, BCPAP, KTC-1, 8305C and BHT-101 cell lines are BRAFV600E mutant thyroid cancer cell lines. TPC-1 and C643 cell lines are non-BRAFV600E mutant thyroid cancer cell lines. All cell lines used in the experiments were purchased from the American Type Culture Collection. All cell lines were identified by short tandem repeat (STR) analysis. All cells were routinely cultured using RPMI 1640 or DMEM (Gibco, Thermo Fisher Scientific) supplemented with 10% fetal bovine serum (Gibco, Thermo Fisher Scientific), 1% nonessential amino acids (Gibco Thermo Fisher Scientific) and 1% penicillin/streptomycin (5,000 units / mL, Gibco, Thermo Fisher Scientific) at 37°C with 5% CO_2_. The highly selective BRAF inhibitor vemurafenib (Selleck Chemicals, Cat #S1267) and the SHP2 inhibitor SHP099 (Selleck Chemicals, Cat #S6388) were dissolved in dimethyl sulfoxide (DMSO). They were added to the medium at the indicated concentrations and diluted during the experiment.

### Plasmid and cell transfection

The lentiviral plasmid for SHP2 knockdown (shSHP2, Cat No GV298, U6-MCS -IRES-puromycin vector) was purchased from Shanghai Gene Chemistry Co. The shRNA sequence targeting SHP2 was as follows: 5'-gcTGAAATAGAAAGCAGAGTT-3'. Lentivirus production was achieved by co-transfection of shRNA, PAX8 and PVSVG into 293T cells using Lipo2000. The virus was collected and filtered 48 h after transfection. Cells were inoculated in 6-well plates at a density of 50,000 cells/well. For transfection, cells were cultured in RPMI 1640 or DMEM containing 10% fetal bovine serum and lentiviral vector for 48 h and then transferred to medium containing 10% fetal bovine serum and 4 μM puromycin for further screening. Successful transfection was then tested by real-time quantitative PCR (RT-qPCR) and western blot.

### IC50 determination

Thyroid cancer cells were inoculated in 96-well plates at 1000 cells/200 µL per well. After the cells were well attached, 10 drug concentration gradients (0, 1, 2, 10, 20, 40, 60, 80, 100, 120 µM) were used for dosing, and the cells were treated in a cell incubator for 96 h. The medium was then aspirated and fresh 1640 or DMEM with CCK8 solutions at a 100:10 ratio was added to 96-well plates which were then incubated at 37°C for 2h. The absorbance was measured with an enzyme-labeled instrument (Thermo) at 450 nm.

### Cell proliferation capacity assay

Thyroid cancer cells were inoculated in 96-well plates at 1000 cells/200 µL per well. After the cells were well attached, they were incubated with DMSO, 5 µM vemurafenib, 10 µM SHP099, or the combination of 5 µM vemurafenib and 10 µM SHP099 for 24 h. Then, the medium was then aspirated, and the cells were incubated with the normal medium for 0 h, 24 h, 48 h, 72 h and 96 h. The medium was then aspirated and fresh 1640 or DMEM with CCK8 solutions at a 100:10 ratio was added to 96-well plates which were then incubated at 37°C for 2 h. The absorbance was measured with an enzyme-labeled instrument (Thermo) at 450 nm.

### Colony formation assay

Thyroid cancer cells were inoculated in 24-well plates at 400 cells/500 µL per well. After the cells were well attached, they were incubated with DMSO, 5 µM vemurafenib, 10 µM SHP099, or the combination of 5 µM vemurafenib and 10 µM SHP099 for 24 h. The medium was then replaced with normal medium for maintenance for 14 days. Colonies were fixed in 4% paraformaldehyde and then stained with 0.1% crystalline violet for observation and counting.

### Cell cycle analysis

Thyroid cancer cells were inoculated in 6-well plates at 5 × 10^5^ cells/2 mL per well. After the cells were well attached, they were incubated with DMSO, 5 µM vemurafenib, 10 µM SHP099, or the combination of 5 µM vemurafenib and 10 µM SHP099 for 24 h. The cells from the different treatments above were trypsin digested, washed with PBS twice and then incubated overnight at 4 °C in 70% ethanol for fixation. The next day, after washing the cells with PBS, the cells were incubated in PBS containing 100 µg/mL RNase A and 5 µg/mL PI (propidium iodide) for 15 min at room temperature and protected from light. Samples were then analyzed using a FACSCalibur flow cytometer (BD Biosciences).

### Western blot

The indicated cells were incubated with DMSO, 5 µM vemurafenib, 10 µM SHP099, or the combination of 5 µM vemurafenib and 10 µM SHP099 for 0 h, 6 h, 24 h and 48 h. The cells were then lysed in RIPA lysis solution containing protease inhibitors for 30 min, and the protein concentration was determined using BCA reagent. Equal amounts of protein were separated by SDS-PAGE and transferred to PVDF membranes and incubated overnight at 4 °C with primary antibodies. The next day, co-incubation with the secondary antibody of the corresponding species was carried out for 2 h. Chemiluminescence detection was performed using the ECL Protein Blotting Assay Kit (Human IgG) (Solarbio). The primary antibodies were used: anti-SHP2 (Cell Signaling Technology, CAT #3397), anti-p-SHP2 (Cell Signaling Technology, CAT #3751), anti-ERK (Cell Signaling Technology, CAT #4695), anti-p-ERK (Cell Signaling Technology, CAT #4370), anti-AKT (Cell Signaling Technology, CAT #4685), anti-p-AKT (Cell Signaling Technology, CAT #4060), anti-MEK (Cell Signaling Technology, CAT #4694), anti-p-MEK (Cell Signaling Technology, CAT #9127), anti-GAPDH (Cell Signaling Technology, CAT #5174).

### RT-qPCR

Cells were collected, and washed twice with prechilled PBS. Then the thyroid cancer cells RNA was isolated and extracted using TRIzol reagent (Invitrogen, Carlsbad, CA, USA). Reverse transcription was performed using Prime Script RT Master Mix (TaKaRa, Tokyo, Japan). Real-time quantitative PCR (RT-qPCR) was performed using premixed SYBR green (TaKaRa, Tokyo, Japan) and specific primers according to the manufacturer's recommendations. Reactions were performed on an ABI 7500 FAST instrument. The primer sequences used for RT-qPCR were shown in **Table S1**.

### Animal experiments

For in vivo studies, vemurafenib and SHP099 were dissolved in 1% hydroxypropylmethylcellulose (HMC) (Sigma) and administered by gavage. A mouse xenograft model was constructed by subcutaneously injecting the KTC-1 cell line (1 × 10^6^) into the right inguinal region of nude mice (Spelford, Beijing, China). When the tumor diameter grew to 5 mm, 20 mice were randomly and equally divided into four groups: the control (HMC) group, which was treated with HMC, the SHP099 group, which was treated with 30 mg/kg SHP099, the vemurafenib group, which was treated with 20 mg/kg vemurafenib, and the combination group, which was treated with the 30 mg/kg SHP099 and 20 mg/kg vemurafenib. The different experimental groups were treated by gavage, with a frequency of once a day. Treatment was administered continuously for 12 days, and body weight and tumor volume were measured every other day (volume = width x length x width/2). The animal experiments involved in the project has been reviewed and approved by The Animal Ethical and Welfare Committee of Tianjin Medical University Cancer Institute and Hospital (IRB Approval No: 2020100).

### H&E staining and immunohistochemistry

Xenograft tumor tissues were embedded in paraffin and sectioned at a thickness of 4 µm. IHC was performed according to standard protocols to quantify Ki67 expression levels to assess the proliferative capacity of the cells. Antibodies used for immunohistochemical detection were mainly Ki67 (Cell Signaling Technology, CST #9449) at a dilution of 1:100.

### Drug-resistant strain culture

Parental cells were treated with vemurafenib at a starting concentration of 10 µM, and after the cells had grown stably at this concentration, the drug concentration was increased. Culturing was continued, with the drug being added in increasing concentrations. Drug induction took 3-5 months until the cells were able to grow stably at the final concentration.

### Statistical analysis

Statistical analysis was performed using the SPSS version 17.0 software and GraphPad Prism 7.0 software. All values are expressed as the mean ± standard deviation (SD). *P* < 0.05 is considered statistically significant. Each experiment was carried out and calculated in triplicate.

## Results

### Negative feedback inhibition of the MAPK signaling pathway is relieved with vemurafenib

**Figure 1:**
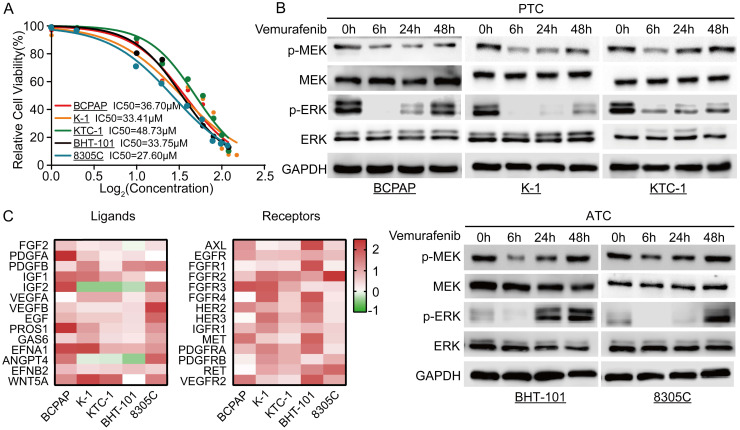
Resistance of thyroid cancer to vemurafenib is associated with upregulation of RTK receptors and ligands. **A** Vemurafenib IC50 detection by CCK-8 assay in BCPAP, K-1, 8305C, BHT-101, and KTC-1 cell lines. **B** Western blot analysis revealed reactivation of the ERK signaling pathway in 5 BRAF mutant thyroid cancer cell lines after 48 h of vemurafenib (5 μM) treatment. **C** Heat map analysis of RT-qPCR results showed activation of multiple RTK receptors and ligands in 5 BRAF mutant thyroid cancer cell lines (BCPAP, K-1, 8305C, BHT-101, and KTC-1) after 48 h of vemurafenib treatment (5 μM).

To determine the optimal dosing concentration of vemurafenib in different BRAFV600E mutant thyroid cancer cell lines, we first determined the IC50 of vemurafenib in different cell lines (Figure 1A). In addition, we also examined the phosphorylation levels of SHP2 in different thyroid cancer cell lines. The results showed that the level of SHP2 phosphorylation was lower in cell lines with BRAFV600E mutation (Figure S1). After vemurafenib administration, we examined the changes in phosphorylation levels of MEK and ERK in the MAPK/ERK signaling pathway at different time points immediately. The results showed that the phosphorylation levels of MEK and ERK in BRAFV600E mutant thyroid cancer cell lines were significantly inhibited after 6 h of treatment with vemurafenib. However, after 48 h, the levels of p-MEK and p-ERK rebounded (Figure 1B). To further determine the mechanism of the rebound of p-MEK and p-ERK levels after vemurafenib administration, we examined the changes in RTK receptors and ligands expression levels by RT-qPCR. The results showed that the expression levels of most RTK receptors and ligands were significantly increased after thyroid cancer cells developed resistance to vemurafenib (Figure 1C). These results suggest that vemurafenib transiently inhibits the activation of the MAPK/ERK signaling pathway in thyroid cancer.

### Blockade of SHP2 significantly enhances sensitivity to vemurafenib

**Figure 2:**
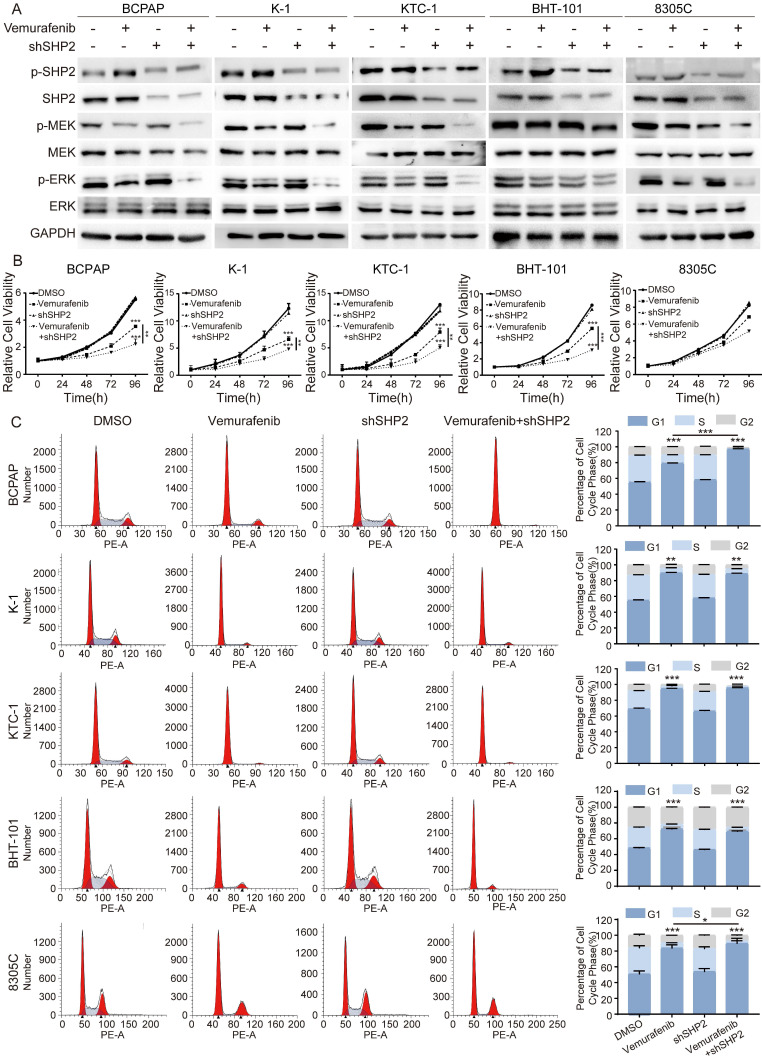
Knockdown of SHP2 expression eliminated ERK rebound after 48 h of vemurafenib treatment. **A** Analysis by western blotting within 48 h of 5 BRAF mutant thyroid cancer cell lines for activation of MAPK signaling pathway. **B** Cell viability detection by CCK-8 assay in 5 BRAF mutant thyroid cancer cell lines. **C** The cell cycle distribution detection by flow cytometric assay in 5 BRAF mutant thyroid cancer cell lines. 5 BRAF mutant thyroid cancer cell lines were treated with DMSO, shSHP2, vemurafenib (5 μM) or shSHP2 + vemurafenib, respectively. *P < 0.05, **P < 0.01, ***P < 0.001. All data are expressed as mean ± SD of three independent experiments.

A growing number of studies have demonstrated that SHP2 activation is a novel mechanism for RTKs to drive cancer progression. Given the critical role of SHP2 in the downstream activation of RTKs, we speculate that SHP2 may play an important role in the process of vemurafenib resistance. We first treated shNC and shSHP2 stable cell lines with DMSO and vemurafenib, respectively, and assayed the activation of the MAPK signaling pathway after 48 h of culture. The results showed that the use of vemurafenib in stable cell lines with SHP2 knockdown consistently inhibited the phosphorylation levels of MEK and ERK compared to the shNC group treated with vemurafenib (Figure 2A, Figure S2). Next, we used CCK8 assays to verify whether blocking SHP2 expression would have a synergistic effect with vemurafenib to inhibit the proliferative ability of thyroid cancer cells. As shown in Figure 2B, with vemurafenib treatment, the proliferation viability of thyroid cancer cells was more significantly inhibited in shSHP2-stable cell lines than that in the shNC group treated with vemurafenib. Inhibition of the proliferative capacity of cancer cells is usually associated with cell cycle arrest. We treated shNC and shSHP2 stable cell lines with DMSO and vemurafenib, respectively, and then investigated the effect on their cell cycles by flow cytometry. Treatment with vemurafenib resulted in increased G1/S phase cycle block in BRAFV600E mutant thyroid cancer cells compared to the shNC group. In addition, treatment of shSHP2 stable cell lines with vemurafenib resulted in a more significant increase in G1/S phase cycle block (Figure 2C). These results suggest that SHP2 may be a key target for alleviating vemurafenib resistance.

### Combination of SHP2 inhibitor and vemurafenib inhibits progression of BRAFV600E mutant thyroid cancer cells

**Figure 3:**
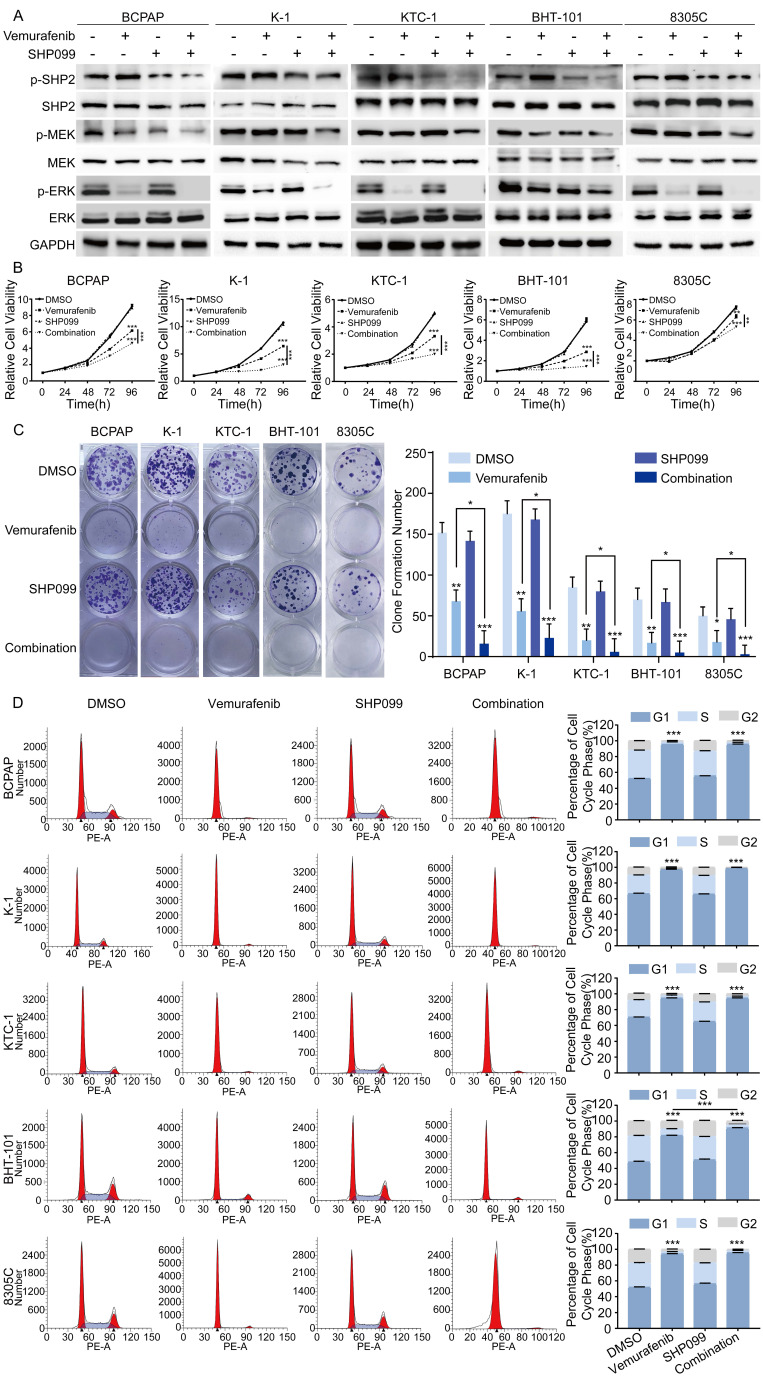
The combination of SHP099 and vemurafenib exhibited synergistic effects in BRAF mutant thyroid cancer cell lines. **A** Analysis by western blotting within 48 h of 5 BRAF mutant thyroid cancer cell lines for activation of MAPK signaling pathway. **B** Cell viability detection by CCK-8 assay in 5 BRAF mutant thyroid cancer cell lines. **C** Cell proliferation detection by colony-formation assay in 5 BRAF mutant thyroid cancer cell lines. **D** The cell cycle distribution detection by flow cytometric assay in 5 BRAF mutant thyroid cancer cell lines. 5 BRAF mutant thyroid cancer cell lines were treated with DMSO, SHP099 (10 μM), vemurafenib (5 μM) or combination (SHP099 + vemurafenib), respectively. *P < 0.05, **P < 0.01, ***P < 0.001. All data are expressed as mean ± SD of three independent experiments.

Considering that SHP2 plays a critical role in the progression of BRAFV600E mutant thyroid cancer, vemurafenib in combination with small molecule inhibitors targeting SHP2 may be a promising therapeutic strategy for the treatment of BRAFV600E mutant thyroid cancer. SHP099 is a highly selective SHP2 variant inhibitor identified in a prior study that showed promising clinical results. To determine whether SHP2 inhibitor were effective in alleviating vemurafenib resistance, we first treated BRAFV600E mutant cell lines with DMSO, vemurafenib, SHP099, and the combination of vemurafenib and SHP099. The expression levels of SHP2, MEK, ERK and their phosphorylation levels were measured after 48 hours. The results showed that the combination of SHP099 and vemurafenib significantly reduced the phosphorylation levels of SHP2, MEK, ERK and significantly enhanced the tumor suppressive effect of vemurafenib compared to that of SHP099 or vemurafenib alone (Figure 3A). Furthermore, we examined the effects of the combination of SHP099 and vemurafenib on the proliferation, colony formation and cycle progression of BRAFV600E mutant thyroid cancer cell lines. The combination of SHP099 and vemurafenib significantly inhibited the proliferation and colony-forming capacity of BRAFV600E mutant thyroid cancer cells compared to SHP099 or vemurafenib alone (Figure 3B-3C). In addition, the combination of SHP099 and vemurafenib also resulted in a significant increase in G1/S phase cycle block compared to SHP099 or vemurafenib alone. A strong growth inhibitory effect was demonstrated (Figure 3D). However, vemurafenib is not effective in non-BRAF mutant thyroid cancer cell lines (Figure S3A-3B). These data suggest that the combination of a SHP2 inhibitor and vemurafenib significantly inhibits the progression of BRAFV600E mutant thyroid cancer cell lines.

### SHP099 enhances the antitumor effects of vemurafenib by alleviating the reactivation of MAPK/ERK signaling

**Figure 4:**
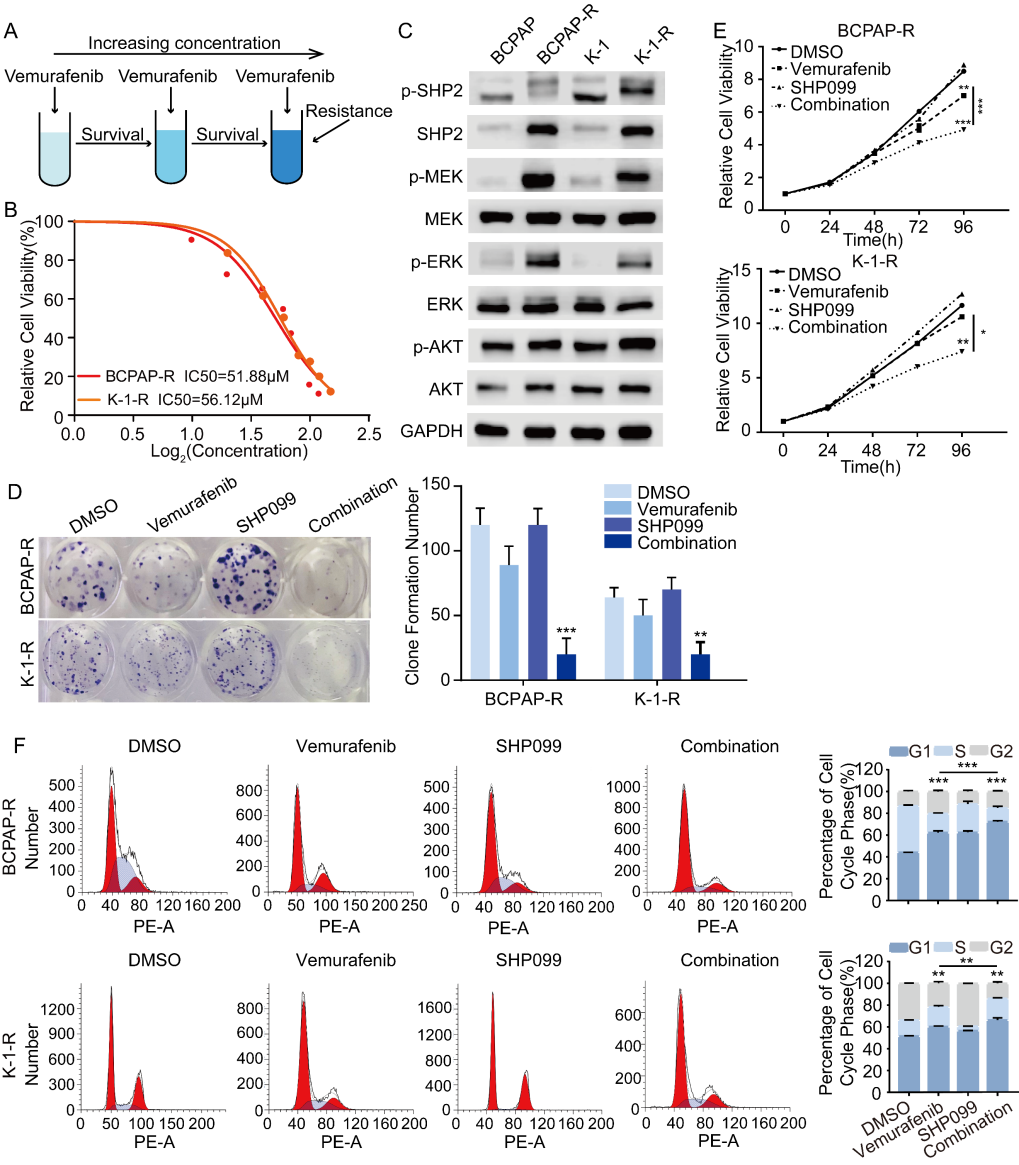
SHP2 is a potential therapeutic target for vemurafenib-resistant thyroid cancer cell lines. **A** Simplified diagram of the construction process of drug-resistant cell lines. **B** Vemurafenib IC50 detection by CCK-8 assay in BCPAP-R and K-1-R cells. **C** SHP2 was significantly activated in BCPAP-R and K-1-R cell lines were significantly activated. Analysis by western blotting within 48 h of two vemurafenib resistance models were treated with DMSO, SHP099 (10 μM), vemurafenib (5 μM) or combination (SHP099 + vemurafenib) for activation of MAPK signaling pathway. **D** Cell proliferation detection by colony-formation assay in BCPAP-R and K-1-R cell lines. **E** Cell viability detection by CCK-8 assay in BCPAP-R and K-1-R cell lines.** F** The cell cycle distribution detection by flow cytometric assay in BCPAP-R and K-1-R cell lines. BCPAP-R and K-1-R cell lines were treated with DMSO, SHP099 (10 μM), vemurafenib (5 μM) or combination (SHP099 + vemurafenib), respectively. *P < 0.05, **P < 0.01, ***P < 0.001. All data are expressed as mean ± SD of three independent experiments.

To further investigate the mechanisms underlying vemurafenib resistance, we constructed models of vemurafenib resistance in vitro using BCPAP and K-1 cell lines. The 2 cell lines were cultured with a high-dose drug shock method under 10 μM concentration while increasing the concentration of vemurafenib until the susceptibility of the resistant clones to vemurafenib was reduced to 2 times the susceptibility of their parental lineage (Figure 4A). We then determined the IC50 of vemurafenib in the 2 resistant pairs (Figure 4B). Previous studies have demonstrated that reactivation of the MAPK/ERK pathway and feedback activation of the PI3K/AKT pathway during the use of vemurafenib can lead to drug resistance in thyroid cancer cells 23. Therefore, we examined changes in the expression levels of SHP2, MEK, ERK, AKT and their phosphorylation levels in BCPAP and K-1 parental cells and drug-resistant cells. The results showed that the phosphorylation levels of SHP2, MEK, ERK and AKT were significantly higher in the resistant cell lines than in the parental cell lines, which confirmed that resistant cell lines had been successfully established (Figure 4C). To further determine the role of SHP2 in promoting vemurafenib resistance, we treated BRAFV600E mutant thyroid cancer cell resistant strains (BCPAP-R and K-1-R) with DMSO, vemurafenib, SHP099, and the combination of vemurafenib and SHP099. The results showed that the combination of SHP099 and vemurafenib effectively inhibited the colony-forming ability, growth ability and cycle progression of the resistant strains (Figure 4D-4F). These data suggest that blocking the expression of SHP2 enhances the sensitivity of BRAFV600E mutant thyroid cancer cell lines that are resistant to vemurafenib. Thus, reactivation of the MAPK/ERK pathway is intrinsic to the development of resistance to vemurafenib, and SHP2, a key protein downstream of RTK signaling, synergistically promotes resistance to vemurafenib in thyroid cancer.

### SHP2 inhibitor enhances the antitumor effect of vemurafenib in vivo

**Figure 5:**
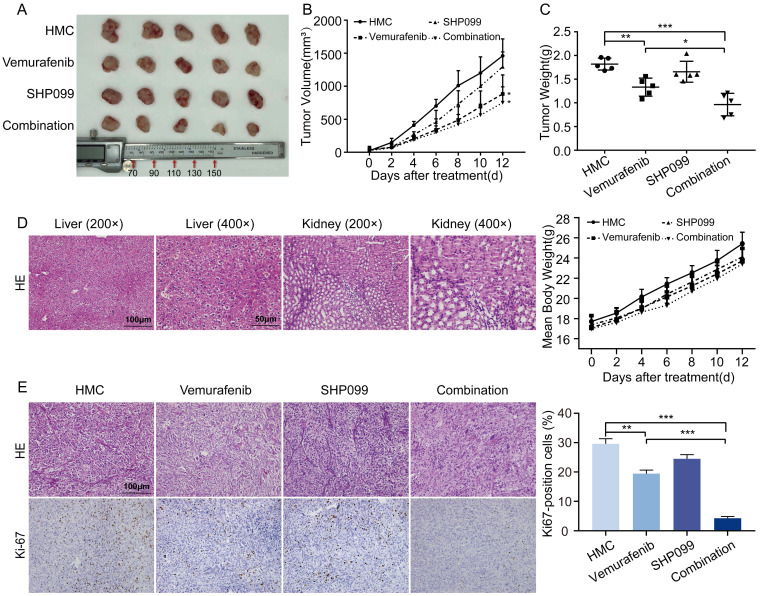
The early combination of vemurafenib and SHP099 is a relatively promising in vivo treatment strategy. **A** Representative image of dissected subcutaneous tumors in mice after 12 days of treatment with HMC, vemurafenib, SHP099 or combination (vemurafenib + SHP099). **B** Changes in tumor volume after 12 days of treatment with HMC, vemurafenib, SHP099 or combination (vemurafenib + SHP099). **C** Tumors weight of dissected subcutaneous tumors in mice after 12 days of treatment with HMC, vemurafenib, SHP099 or combination (vemurafenib + SHP099). **D** Representative H&E staining of liver and kidney after combination (vemurafenib + SHP099) treatments (left). Changes in body weight of mice measured every 2 days after different treatments (right). **E** Representative H&E staining of tumors and representative immunohistochemical staining of Ki67 after different treatments (left). Ki67 expression rate between tumor samples after 12 days of treatment with HMC, vemurafenib, SHP099 or combination (vemurafenib + SHP099) (right).

To determine the antitumor effects of the combination of a SHP2 inhibitor and vemurafenib in vivo, a xenograft mouse model was constructed. Mice treated with vemurafenib alone showed diminished tumor growth capacity compared to HMC group, and mice treated with SHP099 alone showed no significant change in tumor growth capacity. In mice treated with the combination of vemurafenib and SHP099, the tumor growth capacity was significantly inhibited (Figure 5A). In addition, the combination of SHP099 and vemurafenib resulted in a significant reduction in tumor volume and weight compared to treatment with vemurafenib alone (Figure 5B-5C). Meanwhile, we evaluated the toxic effects of the combination of vemurafenib and SHP099. Histopathological results obtained by H&E staining confirmed that the combination of SHP099 and vemurafenib did not cause more severe organ damage in mice compared to treatment with vemurafenib or SHP099 alone, and had no effect on body weight changes in mice (Figure 5D). To quantitatively assess the proliferation index of xenograft tumors, Ki67 expression staining was performed on tumor sections. The number of Ki67-positive cells was lower in the vemurafenib group than in the HMC group and the number of Ki67-positive cells was lower in the vemurafenib and SHP099 combination group than in either single agent treatment group (Figure 5E). Thus, our data demonstrate the efficacy and safety of the combination of vemurafenib and SHP099 for BRAFV600E mutant thyroid cancer treatment.

## Discussion

Most targeted therapies play a role by inhibiting known oncogenic mechanisms in thyroid cancer development and progression. The MAPK signaling pathway is one of the most extensively studied pathways in oncology and is widely associated with different subtypes of thyroid cancers 29, 30. Although multiple kinase inhibitors targeting the MAPK pathway have some clinical benefit, their intrinsic resistance mechanisms and the systemic toxicity of the drugs limit their clinical benefit 31-33. Tumors evade targeted therapies through a wide range of resistance mechanisms. A common mechanism is RTK activation, which occurs by inducing the expression of RTKs and/or their ligands, thereby mediating reactivation of the downstream MAPK and PI3K pathways 34-40. The results of our assays revealed multiple different sets of RTK receptors and ligands activated in response to BRAF inhibitors treatment. We hypothesize that the resistance of PTC and ATC to BRAF inhibitors can be mediated by multiple RTKs. Therefore, combining BRAF and single RTK inhibition may not be a viable therapeutic approach. However, strategies that effectively block signals from multiple activated RTKs may prevent adaptive resistance.

Previously reported clinical studies of vemurafenib in melanoma patients with BRAFV600E mutations provided evidence of high response rates and duration of response to vemurafenib, with overall response rates (ORR) of up to 50%-70% 41-45. Although vemurafenib has demonstrated some promising clinical activity in patients with BRAFV600E-expressing PTC, responses are less common and less impressive than responses observed in BRAFV600E melanoma, with an ORR of only 30%-40% 5, 46.The MAPK and PI3K pathways are downstream signaling pathways of RTKs, and BRAF inhibitors have limited effects on the reactivated PI3K/AKT pathway. Our data show that vemurafenib exhibits only transient inhibitory activity against BRAFV600E mutant thyroid cancer cells. We treated thyroid cancer cells with vemurafenib for 1 h, 6 h, 24 h, 48 h respectively and measured the phosphorylation levels of ERK, MEK and AKT. The results showed that the phosphorylation levels of ERK, MEK and AKT were elevated with the extension of drug treatment time. These results support that the reactivation of the MAPK/ERK signaling pathway and PI3K/AKT signaling pathway limited the effect of vemurafenib on thyroid cancer. SHP2 is signaled downstream of normal RTKs and acts between RTK and RAS 47. SHP099, a potent and specific inhibitor of SHP2, has been developed to block ERK activation and cancer cells proliferation driven by overexpressed, overactivated RTKs 25, 28. Previous studies found that PTPN11 shRNA or CRISPR/cas9-mediated deletion prevented adaptive resistance to vemurafenib in BRAF mutant colon cancer 22. Our findings in PTC and ATC also confirm this conclusion. In our study, it was demonstrated that an SHP2 inhibitor enhanced the sensitivity of BRAFV600E mutant thyroid cancer cells to vemurafenib. Vemurafenib in combination with SHP099 synergistically inhibited proliferation, colony formation and tumorigenic capacity and increased cell cycle arrest in BRAFV600E mutant thyroid cancer cells. Our results suggest that SHP099 can alleviate vemurafenib-induced reactivation of the MAPK and PI3K signaling pathways, with the consequent blockade of adaptive resistance. Therefore, combining vemurafenib with SHP099 may be a promising strategy to alleviate vemurafenib resistance in thyroid cancer.

Although the antitumor effect of BRAF inhibitors in melanoma is significant 41, BRAFV600E mutant thyroid and colorectal cancer cell lines are insensitive to BRAF inhibitors 8, 21, 22. The basis for the specific differences in response to BRAF inhibitors across tumors remains an active area of research. Treatment of colorectal and thyroid cancers with BRAF inhibitors leads to excessive activation of RAS and rapid rebound of ERK signaling, which is more pronounced than that observed in melanoma. These differences may be due to differences in the levels of basic RTK signaling, other mutational features that coexist with BRAF, and specific factors that can lead to resistance to RAF inhibitors 48. One of the most important mechanisms of resistance is the paradoxical activation of ERK followed by the constituent activation of RAF dimers after BRAF inhibitors are used 49. This also implies that BRAF inhibitors are most effective in cells with low levels of RAS activation, in which case the RAF protein is mainly present in monomeric form. We consider that thyroid cancer may have a higher proportion of RAF dimers at baseline than melanoma. Furthermore, the low-level basal RAS activation characteristic in BRAF mutant cells is mediated by the ability of ERK to directly phosphorylate and inhibit various signaling intermediates (e.g., EGFR and SOS) on the one hand, and by the ability of ERK to activate transcription factors that regulate the expression of negative feedback regulators (e.g., SPRY and DUSP) on the other hand 50. Thus, the duration and impact of indirect, transcription-mediated feedback depends on the half-life of the particular factor involved. Furthermore, the impact of feedback is key to how early or late drug resistance occurs. Ligand-induced ERK activation in most RAF inhibitor-sensitive melanoma cells occurs only after RAF inhibition, by which time the feedback factor has been degraded 49. The presence of these differential factors leads to adaptive resistance in thyroid cancer at the early stage of treatment with BRAF inhibitors, unlike melanoma, which develops adaptive resistance to BRAF inhibitors at a later stage. Thus, patients with PTC and ATC should be treated with BRAF inhibitors early with a combination drug strategy to prevent the development of adaptive resistance.

The present study also has some limitations. For drug evaluation, the use of primary human tumor cell lines or patient-derived tumor tissue xenografts in vivo may be more effective for testing treatment regimens 51, 52. However, due to our lack of clinical data from patients treated with vemurafenib, it was not possible to assess the time to development of adaptive resistance to vemurafenib in patients during the actual administration of the drug. Also, the clinical effects of the combination of vemurafenib and SHP099 is unknown. In addition, vemurafenib showed good clinical results in melanoma, but in this research, we did not validate its effects in melanoma cells. Ultimately, extensive clinical trials may be needed to determine the safety and efficacy of the combination of vemurafenib and SHP099 in the treatment of thyroid cancer.

Overall, we found that SHP099 sensitized BRAFV600E mutant thyroid cancer cells to vemurafenib by relieving the reactivation of the MAPK/ERK and PI3K/AKT pathways. These preliminary results suggest that early intervention with SHP099 and vemurafenib combination therapy may be an inspiring therapeutic approach for curing BRAFV600E thyroid cancer.

## Conclusion

In this study, we found that inhibition of SHP2 expression enhanced the sensitivity of thyroid cancer cells to vemurafenib. Vemurafenib in combination with the SHP2 inhibitor SHP099 enhanced the anti-tumor capacity of vemurafenib by slowing down the reactivation of MAPK/ERK and PI3K/AKT signaling pathways. This approach is a potential strategy for the treatment of BRAF mutant thyroid cancer.

## Supplementary Material

Supplementary figures and table.

## Abbreviations

PTC: papillary thyroid cancer
ATC: anaplastic thyroid cancer
RTKs: receptor tyrosine kinases
SHP2: Protein tyrosine phosphatase 2
MAPK: mitogen-activated protein kinase
PTP: protein tyrosine phosphatase
FGF2: fibroblast growth factor 2
PDGFA: platelet-derived growth factor alpha polypeptide
PDGFB: platelet-derived growth factor beta polypeptide
IGF2: insulin-like growth factor 2
VEGFA: vascular endothelial growth factor A
VEGFB: vascular endothelial growth factor B
EGF: epidermal growth factor
GAS6: Growth Arrest-Specific gene 6
alpha): PROS1, protein
IGF1: insulin-like growth factor 1
EFNA1: ephrin-A1
ANGPT4: angiopoietin 4
EFNB2: ephrin-B2
WNT5A: wingless-type MMTV integration site family, member 5A
AXL: AXL receptor tyrosine kinase
FGFR1: fibroblast growth factor receptor 1
FGFR2: fibroblast growth factor receptor 2
FGFR3: fibroblast growth factor receptor 3
FGFR4: fibroblast growth factor receptor 4
HER2: Human epidermal growth factor receptor-2
HER3: Human epidermal growth factor receptor-3
IGFR1: insulin like growth factor 1 receptor
MET: mesenchymal-epithelial transition factor
PDGFRA: platelet—derived growth factor receptor alpha
PDGFRB: platelet derived growth factor receptor beta
RET: proto-oncogene tyrosine-protein kinase receptor ret
VEGFR2: Vascular endothelial growth factor receptor

## References

[1] Cancer Genome Atlas Research N (2014). Integrated genomic characterization of papillary thyroid carcinoma. Cell.

[2] Pozdeyev N, Gay LM, Sokol ES, Hartmaier R, Deaver KE, Davis S (2018). Genetic Analysis of 779 Advanced Differentiated and Anaplastic Thyroid Cancers. Clin Cancer Res.

[3] Landa I, Ibrahimpasic T, Boucai L, Sinha R, Knauf JA, Shah RH (2016). Genomic and transcriptomic hallmarks of poorly differentiated and anaplastic thyroid cancers. J Clin Invest.

[4] Xing M, Westra WH, Tufano RP, Cohen Y, Rosenbaum E, Rhoden KJ (2005). BRAF mutation predicts a poorer clinical prognosis for papillary thyroid cancer. J Clin Endocrinol Metab.

[5] Kim KB, Cabanillas ME, Lazar AJ, Williams MD, Sanders DL, Ilagan JL (2013). Clinical responses to vemurafenib in patients with metastatic papillary thyroid cancer harboring BRAF(V600E) mutation. Thyroid.

[6] Dadu R, Shah K, Busaidy NL, Waguespack SG, Habra MA, Ying AK (2015). Efficacy and tolerability of vemurafenib in patients with BRAF(V600E) -positive papillary thyroid cancer: M.D. Anderson Cancer Center off label experience. J Clin Endocrinol Metab.

[7] Carneiro RM, Carneiro BA, Agulnik M, Kopp PA, Giles FJ (2015). Targeted therapies in advanced differentiated thyroid cancer. Cancer Treat Rev.

[8] Montero-Conde C, Ruiz-Llorente S, Dominguez JM, Knauf JA, Viale A, Sherman EJ (2013). Relief of feedback inhibition of HER3 transcription by RAF and MEK inhibitors attenuates their antitumor effects in BRAF-mutant thyroid carcinomas. Cancer Discov.

[9] Chen S, Su X, Jiang X, Zhang T, Min I, Ding Y (2020). VCAM-1 Upregulation Contributes to Insensitivity of Vemurafenib in BRAF-Mutant Thyroid Cancer. Transl Oncol.

[10] Zhang Y, Xing Z, Liu T, Tang M, Mi L, Zhu J (2022). Targeted therapy and drug resistance in thyroid cancer. Eur J Med Chem.

[11] Giani F, Russo G, Pennisi M, Sciacca L, Frasca F, Pappalardo F (2019). Computational modeling reveals MAP3K8 as mediator of resistance to vemurafenib in thyroid cancer stem cells. Bioinformatics.

[12] Jeong JH, Oh JM, Jeong SY, Lee SW, Lee J, Ahn BC (2019). Combination Treatment with the BRAF(V600E) Inhibitor Vemurafenib and the BH3 Mimetic Navitoclax for BRAF-Mutant Thyroid Carcinoma. Thyroid.

[13] Wang W, Kang H, Zhao Y, Min I, Wyrwas B, Moore M (2017). Targeting Autophagy Sensitizes BRAF-Mutant Thyroid Cancer to Vemurafenib. J Clin Endocrinol Metab.

[14] Chambard JC, Lefloch R, Pouyssegur J, Lenormand P (2007). ERK implication in cell cycle regulation. Biochim Biophys Acta.

[15] Avruch J, Khokhlatchev A, Kyriakis JM, Luo Z, Tzivion G, Vavvas D (2001). Ras activation of the Raf kinase: tyrosine kinase recruitment of the MAP kinase cascade. Recent Prog Horm Res.

[16] Lito P, Pratilas CA, Joseph EW, Tadi M, Halilovic E, Zubrowski M (2012). Relief of profound feedback inhibition of mitogenic signaling by RAF inhibitors attenuates their activity in BRAFV600E melanomas. Cancer Cell.

[17] Bagheri-Yarmand R, Busaidy NL, McBeath E, Danysh BP, Evans KW, Moss TJ (2021). RAC1 Alterations Induce Acquired Dabrafenib Resistance in Association with Anaplastic Transformation in a Papillary Thyroid Cancer Patient. Cancers (Basel).

[18] Straussman R, Morikawa T, Shee K, Barzily-Rokni M, Qian ZR, Du J (2012). Tumour micro-environment elicits innate resistance to RAF inhibitors through HGF secretion. Nature.

[19] Villanueva J, Vultur A, Lee JT, Somasundaram R, Fukunaga-Kalabis M, Cipolla AK (2010). Acquired resistance to BRAF inhibitors mediated by a RAF kinase switch in melanoma can be overcome by cotargeting MEK and IGF-1R/PI3K. Cancer Cell.

[20] Wilson TR, Fridlyand J, Yan Y, Penuel E, Burton L, Chan E (2012). Widespread potential for growth-factor-driven resistance to anticancer kinase inhibitors. Nature.

[21] Corcoran RB, Ebi H, Turke AB, Coffee EM, Nishino M, Cogdill AP (2012). EGFR-mediated re-activation of MAPK signaling contributes to insensitivity of BRAF mutant colorectal cancers to RAF inhibition with vemurafenib. Cancer Discov.

[22] Prahallad A, Sun C, Huang S, Di Nicolantonio F, Salazar R, Zecchin D (2012). Unresponsiveness of colon cancer to BRAF(V600E) inhibition through feedback activation of EGFR. Nature.

[23] Lito P, Rosen N, Solit DB (2013). Tumor adaptation and resistance to RAF inhibitors. Nat Med.

[24] Hof P, Pluskey S, Dhe-Paganon S, Eck MJ, Shoelson SE (1998). Crystal structure of the tyrosine phosphatase SHP-2. Cell.

[25] Garcia Fortanet J, Chen CH, Chen YN, Chen Z, Deng Z, Firestone B (2016). Allosteric Inhibition of SHP2: Identification of a Potent, Selective, and Orally Efficacious Phosphatase Inhibitor. J Med Chem.

[26] Ostman A, Hellberg C, Bohmer FD (2006). Protein-tyrosine phosphatases and cancer. Nat Rev Cancer.

[27] Grossmann KS, Rosario M, Birchmeier C, Birchmeier W (2010). The tyrosine phosphatase Shp2 in development and cancer. Adv Cancer Res.

[28] Chen YN, LaMarche MJ, Chan HM, Fekkes P, Garcia-Fortanet J, Acker MG (2016). Allosteric inhibition of SHP2 phosphatase inhibits cancers driven by receptor tyrosine kinases. Nature.

[29] Nikiforov YE (2008). Thyroid carcinoma: molecular pathways and therapeutic targets. Mod Pathol.

[30] Naoum GE, Morkos M, Kim B, Arafat W (2018). Novel targeted therapies and immunotherapy for advanced thyroid cancers. Mol Cancer.

[31] Bikas A, Vachhani S, Jensen K, Vasko V, Burman KD (2016). Targeted therapies in thyroid cancer: an extensive review of the literature. Expert Rev Clin Pharmacol.

[32] Widakowich C, de Castro G Jr, de Azambuja E, Dinh P, Awada A (2007). Review: side effects of approved molecular targeted therapies in solid cancers. Oncologist.

[33] Bible KC, Ryder M (2016). Evolving molecularly targeted therapies for advanced-stage thyroid cancers. Nat Rev Clin Oncol.

[34] Manchado E, Weissmueller S, Morris JPt, Chen CC, Wullenkord R, Lujambio A (2016). A combinatorial strategy for treating KRAS-mutant lung cancer. Nature.

[35] Sun C, Hobor S, Bertotti A, Zecchin D, Huang S, Galimi F (2014). Intrinsic resistance to MEK inhibition in KRAS mutant lung and colon cancer through transcriptional induction of ERBB3. Cell Rep.

[36] Anderson GR, Winter PS, Lin KH, Nussbaum DP, Cakir M, Stein EM (2017). A Landscape of Therapeutic Cooperativity in KRAS Mutant Cancers Reveals Principles for Controlling Tumor Evolution. Cell Rep.

[37] Duncan JS, Whittle MC, Nakamura K, Abell AN, Midland AA, Zawistowski JS (2012). Dynamic reprogramming of the kinome in response to targeted MEK inhibition in triple-negative breast cancer. Cell.

[38] Zawistowski JS, Bevill SM, Goulet DR, Stuhlmiller TJ, Beltran AS, Olivares-Quintero JF (2017). Enhancer Remodeling during Adaptive Bypass to MEK Inhibition Is Attenuated by Pharmacologic Targeting of the P-TEFb Complex. Cancer Discov.

[39] Lin L, Sabnis AJ, Chan E, Olivas V, Cade L, Pazarentzos E (2015). The Hippo effector YAP promotes resistance to RAF- and MEK-targeted cancer therapies. Nat Genet.

[40] Nazarian R, Shi H, Wang Q, Kong X, Koya RC, Lee H (2010). Melanomas acquire resistance to B-RAF(V600E) inhibition by RTK or N-RAS upregulation. Nature.

[41] Chapman PB, Hauschild A, Robert C, Haanen JB, Ascierto P, Larkin J (2011). Improved survival with vemurafenib in melanoma with BRAF V600E mutation. N Engl J Med.

[42] Robert C, Karaszewska B, Schachter J, Rutkowski P, Mackiewicz A, Stroiakovski D (2015). Improved overall survival in melanoma with combined dabrafenib and trametinib. N Engl J Med.

[43] Ascierto PA, McArthur GA, Dreno B, Atkinson V, Liszkay G, Di Giacomo AM (2016). Cobimetinib combined with vemurafenib in advanced BRAF(V600)-mutant melanoma (coBRIM): updated efficacy results from a randomised, double-blind, phase 3 trial. Lancet Oncol.

[44] McArthur GA, Chapman PB, Robert C, Larkin J, Haanen JB, Dummer R (2014). Safety and efficacy of vemurafenib in BRAF(V600E) and BRAF(V600K) mutation-positive melanoma (BRIM-3): extended follow-up of a phase 3, randomised, open-label study. Lancet Oncol.

[45] Dummer R, Ascierto PA, Gogas HJ, Arance A, Mandala M, Liszkay G (2018). Overall survival in patients with BRAF-mutant melanoma receiving encorafenib plus binimetinib versus vemurafenib or encorafenib (COLUMBUS): a multicentre, open-label, randomised, phase 3 trial. Lancet Oncol.

[46] Brose MS, Cabanillas ME, Cohen EE, Wirth LJ, Riehl T, Yue H (2016). Vemurafenib in patients with BRAF(V600E)-positive metastatic or unresectable papillary thyroid cancer refractory to radioactive iodine: a non-randomised, multicentre, open-label, phase 2 trial. Lancet Oncol.

[47] Ran H, Tsutsumi R, Araki T, Neel BG (2016). Sticking It to Cancer with Molecular Glue for SHP2. Cancer Cell.

[48] Yang H, Higgins B, Kolinsky K, Packman K, Bradley WD, Lee RJ (2012). Antitumor activity of BRAF inhibitor vemurafenib in preclinical models of BRAF-mutant colorectal cancer. Cancer Res.

[49] Poulikakos PI, Zhang C, Bollag G, Shokat KM, Rosen N (2010). RAF inhibitors transactivate RAF dimers and ERK signalling in cells with wild-type BRAF. Nature.

[50] Lake D, Correa SA, Muller J (2016). Negative feedback regulation of the ERK1/2 MAPK pathway. Cell Mol Life Sci.

[51] Newell DR (2001). Flasks, fibres and flanks-pre-clinical tumour models for predicting clinical antitumour activity. Br J Cancer.

[52] Gao H, Korn JM, Ferretti S, Monahan JE, Wang Y, Singh M (2015). High-throughput screening using patient-derived tumor xenografts to predict clinical trial drug response. Nat Med.

