# Supplementary material for: Early Combined SHP2 Targeting Reverses the Therapeutic Resistance of Vemurafenib in Thyroid Cancer
Source: J Cancer. 2023 May 29;14(9):1592–604. doi: 10.7150/jca.83853 (PMC10266257; doi:10.7150/jca.83853)
Supplement: Supplementary file 1 — Supplementary figures and table. [file jcav14p1592s1.pdf]

Table S1: The primers of RT-qPCR assays

	Forward	Reverse
FGF2	AGCGGCTGTACTGCAAAAACGG	CCTTTGATAGACACAACTCCTCTC
PDGFA	CAGCGACTCCTGGAGATAGACT	CGATGCTTCTCTTCCTCCGAATG
PDGFB	GAGATGCTGAGTGACCACTCGA	GTCATGTTCAGGTCCAACTCGG
IGF1	CTCTTCAGTTCGTGTGTGGAGAC	CAGCCTCCTTAGATCACAGCTC
IGF2	TGGCATCGTTGAGGAGTGCTGT	ACGGGGTATCTGGGGAAGTTGT
VEGFA	TTGCCTTGCTGCTCTACCTCCA	GATGGCAGTAGCTGCGCTGATA
VEGFB	AAGGACAGTGCTGTGAAGCCAG	TGGAGTGGGATGGGTGATGTCA
EGF	TGCGATGCCAAGCAGTCTGTGA	GCATAGCCCAATCTGAGAACCAC
GAS6	CCTTCCATGAGAAGGACCTCGT	GAAGCACTGCATCCTCGTGTTC
PROS1	GGCTCCTACTATCCTGGTTCTG	CAAGGCAAGCATAACACCAGTGC
EFNA1	CTGTCTGAGAAGTTCCAGCGCT	CCACTGACAGTCACCTTCAACC
ANGPT4	GTGGTGTCTACACCATCCAGGT	TCCGCTGAAAATTCACGGTGCC
EFNB2	GCAAGTTCTGCTGGATCAACCAG	GCTGTTGCCGTCTGTGCTAGAA
WNT5A	TACGAGAGTGCTCGCATCCTCA	TGTCTTCAGGCTACATGAGCCG
AXL	GTTTGGAGCTGTGATGGAAGGC	CGCTTCACTCAGGAAATCCTCC
EGFR	AACACCCTGGTCTGGAAGTACG	TCGTTGGACAGCCTTCAAGACC
FGFR1	GCACATCCAGTGGCTAAAGCAC	AGCACCTCCATCTCTTTGTCGG
FGFR2	GTGCCGAATGAAGAACACGACC	GGCGTGTTGTTATCCTCACCAG
FGFR3	TCCATCTCCTGGCTGAAGAACG	TGTTCTCCACGACGCAGGTGTA
FGFR4	AACACCGTCAAGTTCCGCTGTC	CATCACGAGACTCCAGTGCTGA
HER2	GGAAGTACACGATGCGGAGACT	ACCTTCCTCAGCTCCGTCTCTT
HER3	CTATGAGGCGATACTTGGAACGG	GCACAGTTCCAAAGACACCCGA
IGFR1	TCGACATCCGCAACGACTATC	CCAGGGCGTAGTTGTAGAAGAG
MET	TGCACAGTTGGTCCTGCCATGA	CAGCCATAGGACCGTATTTCGG
PDGFRA	GACTTTCGCCAAAGTGGAGGAG	AGCCACCGTGAGTTCAGAACGC
PDGFRB	TGCAGACATCGAGTCCTCCAAC	GCTTAGCACTGGAGACTCGTTG
RET	GTCCTCTTGCTCCACTTCAACG	CCTGGCAGTTTTCCACACAGAC
VEGFR2	GGAACCTCACTATCCGCAGAGT	CCAAGTTCGTCTTTTCCTGGGC

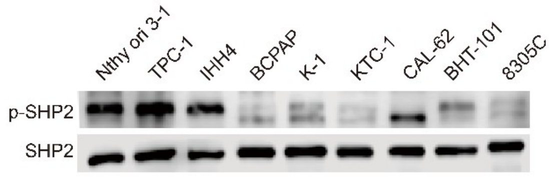

Supplementary Figure 1. Phosphorylation levels of SHP2 were significantly higher in non-BRAFV600E mutant thyroid cancer cell lines than in BRAFV600E mutant thyroid cancer cell lines.

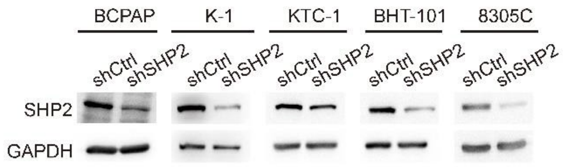

Supplementary Figure 2. Validation of knockdown levels of SHP2 in thyroid cancer cell lines with different BRAFV600E.

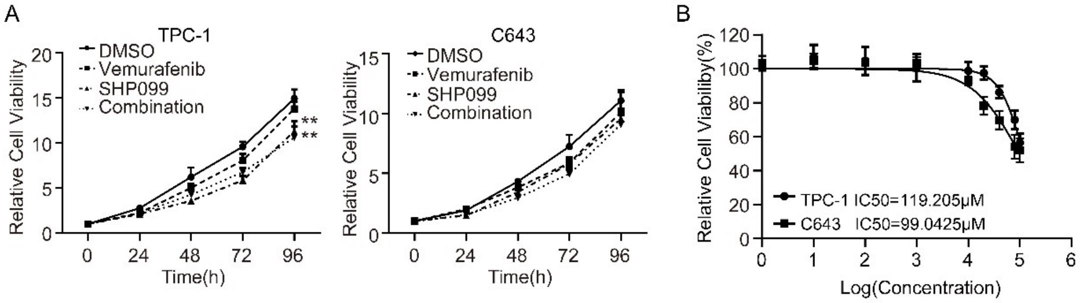

Supplementary Figure 3. The vemurafenib had no effect in non-BRAF mutant thyroid cancer cell lines. **A** Cell viability detection by a CCK-8 assay in TPC-1 and C643 cell lines. **B** Vemurafenib IC50 detection by a CCK-8 assay in TPC-1 and C643 cell lines.
